# Vitamin D signaling regulates oral keratinocyte proliferation *in vitro* and *in vivo*

**DOI:** 10.3892/ijo.2014.2338

**Published:** 2014-03-12

**Authors:** FENG-NING F. YUAN, JAYASANKER VALIYAPARAMBIL, MICHAEL C. WOODS, HUY TRAN, RIMA PANT, JOHN S. ADAMS, SANJAY M. MALLYA

**Affiliations:** 1 Division of Diagnostic and Surgical Sciences, School of Dentistry, University of California, Los Angeles, CA 90095;; 2 University of Connecticut Health Center, School of Dental Medicine, Farmington, CT 06032;; 3 Orthopaedic Hospital Research Center and Department of Orthopaedic Surgery, David Geffen School of Medicine at UCLA, University of California, Los Angeles, CA 90095, USA

**Keywords:** vitamin D, oral cancer, keratinocytes

## Abstract

The secosteroidal hormone 1,25-dihyroxyvitamin D [1,25(OH)_2_D_3_] and its receptor, the vitamin D receptor (VDR), are crucial regulators of epidermal proliferation and differentiation. However, the effects of 1,25(OH)_2_D_3_-directed signaling on oral keratinocyte pathophysiology have not been well studied. We examined the role of 1,25(OH)_2_D_3_ in regulating proliferation and differentiation in cultured oral keratinocytes and on the oral epithelium *in vivo.* Using lentiviral-mediated shRNA to silence VDR, we generated an oral keratinocyte cell line with stable knockdown of VDR expression. VDR knockdown significantly enhanced proliferation and disrupted calcium- and 1,25(OH)_2_D_3_-induced oral keratinocyte differentiation, emphasizing the anti-proliferative and pro-differentiation effects of 1,25(OH)_2_D_3_ in oral keratinocytes. Using vitamin D_3_-deficient diets, we induced chronic vitamin D deficiency in mice as evidenced by decreased serum 25-hydroxyvitamin D (25OHD) concentrations. The vitamin D-deficient mice manifested increased proliferation of the tongue epithelium, but did not develop any morphological or histological abnormalities in the oral epithelium, suggesting that vitamin D deficiency alone is insufficient to alter oral epithelial homeostasis and provoke carcinogenesis. Immunohistochemical analyses of human and murine oral squamous cell carcinomas showed increased VDR expression. Overall, our results provide strong support for a crucial role for vitamin D signaling in oral keratinocyte pathophysiology.

## Introduction

The secosteroidal hormone vitamin D has well-established functions in calcium homeostasis and bone health. In addition, it is now known that vitamin D has important and essential functions in regulating cell proliferation and differentiation, and immunomodulation in a variety of cell types (1). Vitamin D is acquired through the diet or via UV-catalyzed dermal synthesis, and is subsequently converted to its active hormonal form via two sequential hydroxylation reactions. The first reaction occurs in the liver, where vitamin D is converted to its major circulating form, 25-hydroxyvitamin D (25OHD). This form is subsequently hydroxylated, predominantly in the kidney, to its active metabolite, 1,25-dihydroxyvitamin D [1,25(OH)_2_D]. Importantly, the enzyme that catalyzes this latter reaction, CYP27B1, is also expressed at several extra-renal sites (2,3), including dermal keratinocytes (4). It is thought that locally produced 1,25(OH)_2_D may have autocrine or paracrine functions. The effects of vitamin D are mediated via the vitamin D receptor (VDR), a nuclear hormone receptor that functions to activate or repress transcription of specific vitamin D target genes. The VDR forms a heterodimer with the retinoid X receptor (RXR), and binds to vitamin D response elements (VDRE), specific DNA sequences located within the promoters of target genes. Co-regulator complexes link the VDR-RXR heterodimer to the transcriptional machinery and provide an additional regulation in vitamin D-mediated gene transcription (5). In dermal keratinocytes, 1,25(OH)_2_D_3_ inhibits proliferation and enhances differentiation (6). Transcriptional profiling has shown that in this cell type, vitamin D induces genes involved in differentiation, proliferation and immune response (7), underscoring the importance of vitamin D signaling in keratinocyte physiology. Studies of transgenic animal models have further substantiated the importance of vitamin D signaling in keratinocyte pathophysiology. VDRE-null mice demonstrate reduced epidermal differentiation (8), and are sensitive to chemical carcinogen and UV-induced carcinogenesis (9,10), suggesting a potential tumor-suppressive role for VDR (11). Mice with knockout of the CYP27B1 gene also manifest decreased epidermal differentiation (12).

There has been considerable interest in the role and contribution of vitamin D to oral health, including it potential roles in periodontal disease (13) and oral squamous cell carcinoma (OSCC). Cross-sectional epidemiological studies have found an association between low levels of 25-hydroxyvitamin D an increased OSCC risk (14,15). Although the vitamin D effects on dermal keratinocytes have been well studied, its effects on oral keratinocytes are less well understood. Although there are several similarities between the epidermis and the oral mucosal epithelium, there are also evident differences between these two epithelia. Embryologically, the skin is derived from the ectoderm. In contrast, the oral mucosal epithelium may be either ectodermal or endodermal in origin, depending on its anatomic location. Unlike the mesoderm-derived dermis, the connective tissue of the oral mucosa originates from the ectomesenchyme, in particular, from the neural crest cells. The similarities and differences between the epidermis and the oral epithelium are emphasized by the overlap as well as dissimilarity in the expression of differentiation markers and adhesion molecules (16). In this study, we investigated the contribution of VDR-mediated signaling to oral keratinocyte physiology *in vitro,* its deregulation in human and murine OSCC, and examined the vitamin D effects on oral epithelial proliferation *in vivo.*

## Materials and methods

### Cells and culture conditions

OKF6-TERT1 cells (obtained from Dr James Rheinwald, Brigham and Women’s Hospital, Boston, MA) are immortalized, but non-transformed oral keratinocytes (17) that stably express hTERT, the catalytic subunit of telomerase. Cells were grown in keratinocyte serum-free medium (Invitrogen Corporation, Carlsbad, CA), supplemented with bovine pituitary extract (25 *μ*g/ml), epidermal growth factor (0.2 ng/ml), penicillin (100 IU/ml) and streptomycin (100 *μ*g/ml). The calcium concentration of the media was adjusted to 0.03 mM, which maintained the keratinocytes in a proliferative state. Keratinocyte differentiation was induced by increasing the calcium concentration (1.2 mM) or addition of 1,25(OH)_2_D_3_ (10^−8^ M) to the growth media.

### shRNA mediated VDR knockdown

To knock down VDR function, we used three validated lentiviral constructs expressing small hairpin RNA (shRNA) sequences to target three different regions of the human VDR transcript. These constructs, developed by the RNAi Consortium, were obtained from Sigma-Aldrich (St. Louis, MO). Details of the clones and target sequences are provided in Table I. Lentiviral particles were prepared using standard protocols, resuspended in keratinocyte serum-free media and used to transduce OKF6-TERT1 cells. After 48 h, stably transduced cells were selected for puromycin resistance (2.5 *μ*g/ml) for 10 days.

### Cell growth and cell cycle assays

Cells were seeded into 12-well plates at densities of approximately 5,000 cells per well. At 24, 48 and 72 h, the media were aspirated and the plates were stored at −80°C. Cell proliferation was assayed using the Cyquant kit, according to the manufacturer’s instructions (Invitrogen Corporation). Four independent samples from each cell line were analyzed.

To analyze cell cycle distribution, exponentially growing cells were detached using accutase, and centrifuged at 300 × g for 5 min. The cell pellet was resuspended in keratinocyte growth media containing 2 *μ*g/ml of Hoechst 33342 DNA staining reagent (Invitrogen Corporation). DNA content was analyzed on a FACScan flow cytometer (Beckton-Dickinson, Franklin Lakes, NJ). Three independent samples from each cell line were analyzed.

### Western blot analyses

Cells were lysed in radioimmunoprecipitation assay (RIPA) buffer for 1 h on ice. Cell debris was removed by centrifugation at 10,000 × g for 10 min at 4°C. Equal amounts of protein were layered onto reducing SDS-PAGE gels and transferred onto PVDF membranes. The following primary antibodies were used for immunoblotting: mouse monoclonal anti-human cyclin D1 (clone DCS-6), rat anti-chicken VDR (clone 9A7), mouse monoclonal anti-human involucrin (clone SY-5, Sigma-Aldrich), and mouse monoclonal antibody against human GAPDH, as loading control (Abcam Inc., Cambridge, MA). Binding of the primary antibody was detected using the appropriate horseradish peroxidase-conjugated IgG and Luminol reagent (Santa Cruz Biotechnology, Santa Cruz, CA).

### Immunofluorescence

Cells were allowed to attach overnight to glass coverslips in 12-well plates. The calcium concentration of the media was adjusted to 1.2 mM and the cells were incubated for 48 h. Cells were fixed with 4% formaldehyde for 10 min and washed in PBS. To detect E-cadherin, cells were blocked with 5% goat serum and incubated with a rabbit monoclonal anti-human E-cadherin (24E10, Cell Signaling Technology, Beverley, MA) for 2 h at room temperature. Bound primary antibody was detected using an Alexa Fluor 546-conjugated goat anti-rabbit IgG (Invitrogen Corporation) and the nuclei were counterstained with TOPO-3 (Invitrogen Corporation). Sections were examined by confocal microscopy.

### Quantitative real-time PCR

For quantitative PCR analyses, total RNA was extracted using TRIzol reagent (Invitrogen Corporation) and reverse transcribed using random hexamers. The cDNA templates were amplified using gene-specific primers to amplify cyclin D1 and the TATA-binding protein (TBP, as endogenous control). Sequences of the primers are as follows: cyclin D1 (forward, 5′-CAGAGGCGGAGGAGAACAAA-3′; reverse, 5′-ATGGAGGGCGGATTGGAA-3′) and TBP (forward, 5′-CACGAACCACGGCACTGATT-3′, reverse, 5′-TTTTCTTGCTGCCAGTCTGGAC-3′). Real-time PCR reactions were done in triplicate on an ABI 7900HT real-time PCR unit using SYBR-Green detection and fold changes were analyzed using the ΔΔCT method.

### Animals and diets

For the dietary manipulation studies, we used 4-month old adult FVB mice. The animals were initially maintained on a standard rodent diet and subsequently shifted on to a vitamin D-deficient diet (0.05 IU/g cholecalciferol, 0.5% calcium) or a vitamin D-replete diet (1.0 IU/g cholecalciferol, 0.5% calcium). The diets were custom fabricate by Harlan Laboratories (Madison, WI). Mice were maintained on the deficient or replete diets for four months. During the entire experimental period mice were housed with *ad libitum* access to water and food in a controlled-temperature room. Mice were housed in standard cages without shielding from UVB and maintained on a standard 12-h-light/dark cycle. Blood was collected by retro-orbital bleeding, under isoflurane anesthesia, prior to and every month following the start of the dietary manipulations. At the end of the experimental period, blood was collected by terminal cardiac puncture. The oral cavity was examined for any gross morphological alterations and the tongues dissected out, fixed overnight in 10% neutral buffered formalin, transferred to 70% ethanol and embedded in paraffin. Sections (5-*μ*m thick) were obtained from the paraffin-embedded blocks and stained with hematoxylin and eosin.

To induce oral epithelial dysplasia and neoplasia, we used the chemical carcinogen 4-nitroquinoline 1-oxide (4NQO), as previously described (18). A total of 5 C57BL/6 mice, approximately 6 months old, were treated with 4NQO (100 ppm, delivered via the drinking water) for a period of 8 weeks. Following the carcinogen treatment period, mice were fed with normal water for 16 weeks. Through the entire experimental period, mice were fed with standard rodent diets, replete in cholecalciferol. At the end of the experimental period, mice were euthanized and the tongues examined for any gross morphological examinations, and processed for histological examination as described above.

Animal experimentation was reviewed and approved by the institutional animal care and use committees at the University of California (Los Angeles, CA).

### Biochemical measurements

Following collection, blood was separated into serum and used to measure 25OHD_3_, using a commercial ELISA kit according to the manufacturer’s instructions (Immunodiagnostic Systems Inc, Fountain Hills, AZ). Total serum calcium was measured using the cresolphthalein complexone method using a commercial kit according to the manufacturer’s instructions (Stanbio Laboratory, Boerne, TX).

### Tumors and immunohistochemistry

For human tumor analyses, we used de-identified, archival formalin-fixed paraffin-embedded tissues (16 human OSCC and 4 normal oral mucosa). The studies were approved by the Institutional Review Board at the University of California. For the murine tissue analyses, we used paraffin-embedded tissues from mice fed with the vitamin D-replete/deplete diets, and from mice treated with 4NQO as described above.

Tissues were sectioned onto glass slides. Sections were deparaffinized in xylene, cleared through a graded ethanol series and then hydrated in distilled water. For epitope retrieval, slides were heated for 15 min in citrate buffer, pH 6.0, in an electric pressure cooker. Endogenous peroxidase was quenched with 3% H_2_O_2_ and the sections were incubated for 30 min in 5% normal serum to reduce non-specific binding. The following antibodies were used: anti-Ki-67 (Clone Tec3, DakoCytomation, Carpinteria, CA); anti-VDR (Clone 9A7, Affinity Bioreagents, Rockford, IL). Binding of the primary antibodies was detected using the appropriate biotinylated IgG and the avidin-biotin complex (Vector Laboratories, Burlingame, CA) using 3,3-diaminobenzidine as a chromagen (Zymed, Invitrogen Corporation). Sections were counter-stained in hematoxylin.

The labeling index for Ki67 was determined by counting the number of positively immunostained cells using the CellSens software (Olympus, Center Valley, PA) and expressed as positive cells per unit area. Approximately 150–200 cells from three distinct areas were counted on each section. For the human tumor analyses, two observers (SM and RP) independently analyzed the stained sections. For each sample, three separate sections were examined and the intensity of staining was graded on a 4 point scale: 0, no staining; 1, weak staining; 2, moderate staining; and 3, strong staining. The scores from the two observers were averaged.

## Results

### Knockdown of VDR enhances oral keratinocyte proliferation

**Figure 1.**
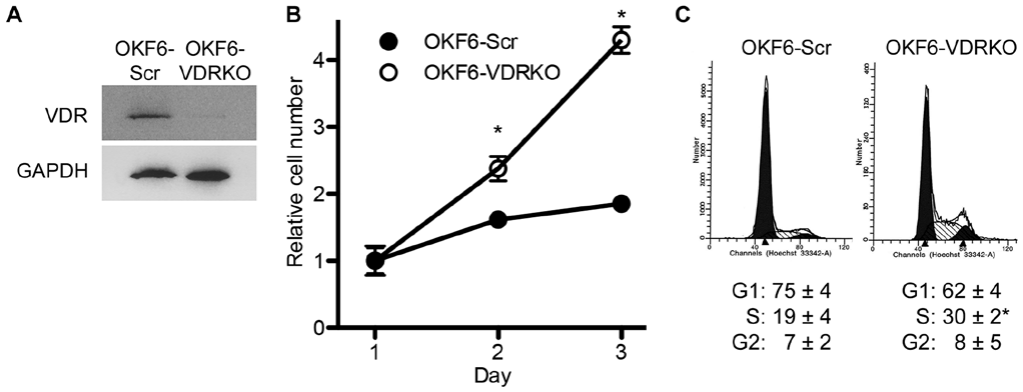
VDR knockdown enhances oral keratinocyte proliferation. (A) OKF6-TERT1 keratinocytes were transduced with lentiviral particles containing either scrambled shRNA (OKF6-Scr) or shRNAs directed against the human VDR transcript (OKF6-VDRKO). Western blot analysis of stably transduced cells demonstrates almost complete absence of VDR protein in OKF6-VDRKO cells. (B) Cell proliferation was significantly enhanced in oral keratinocytes with VDR knockdown (OKF6-VDRKO), compared with control, scrambled shRNA transduced cells (OKF6-Scr). Mean ± SD, ^*^p≤0.001. (C) Cell cycle distribution in OKF6-Scr and OKF6-VDRKO cells. Cells were stained with Hoechst 33342 and analyzed by flow cytometry. Numbers are mean ± SD, ^*^p<0.05 compared with OKF6-Scr.

To examine the role of VDR in regulating oral keratinocyte physiology, we used lentiviral-based shRNA to ablate the function of VDR in OKF6-TERT1 cells. We used three shRNA constructs to target three discrete regions of the human VDR transcript (Table I). Stably transduced cells (OKF6-VDRKO) were enriched by puromycin selection. Western blot analysis confirmed an almost complete absence of VDR protein levels in the VDRKO cells compared with control, scrambled shRNA transduced cells (OKF6-Scr, Fig. 1A).

We assayed cell proliferation in first passage OKF6-VDRKO and OKF6-Scr cells, using the Cyquant cell proliferation assay. VDR knockdown significantly enhanced cell proliferation (Fig. 1B, p≤0.0001). Numbers of VDR knockdown cells increased 2.4-fold and 4.3-fold on days 2 and 3, respectively. In contrast, numbers of scrambled shRNA-transduced control cells increased only 1.6-fold and 1.9-fold for the same time intervals. Analysis of cell cycle distribution showed a significant increase in the proportion of cells in the S phase in OKF6-VDRKO cells (p<0.05, Fig. 1C). Thus, blocking VDR function significantly enhanced cell proliferation.

**Figure 2.**
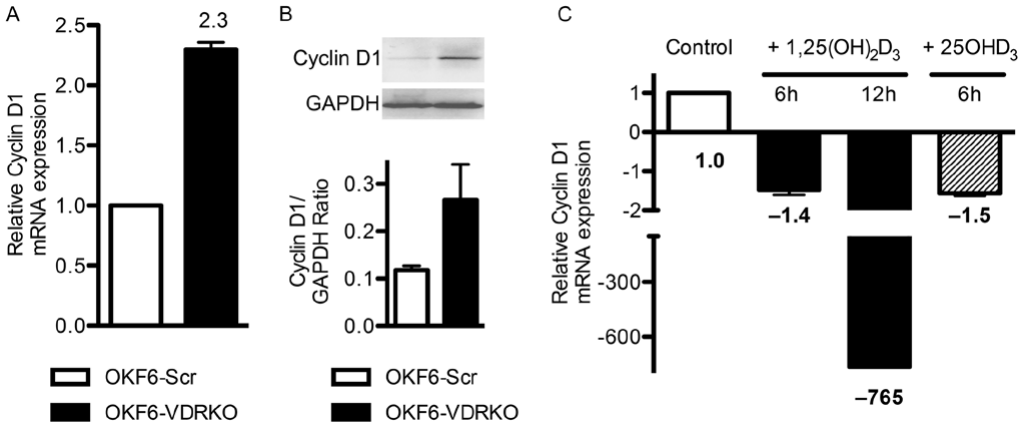
VDR regulates cyclin D1 expression in oral keratinocytes. (A) Quantitative RT-PCR analysis of RNA for cyclin D1 mRNA expression. Total RNA from control (OKF6-Scr) and VDR knockdown cells (OKF6-VDRKO) was isolated and cyclin D1 mRNA quantified by real-time PCR. Cyclin D1 expression was normalized for expression of the housekeeping gene TBP Mean ± SD, ^*^p≤0.0001. (B) Representative western blot analysis for cyclin D1 protein levels. Whole cell lysates were immunoblotted to detect expression of cyclin D1. The levels of cyclin D1 were normalized to GAPDH. The bar graph shows quantitative analysis of cyclin D1 protein levels from 3 independent western blot analyses. Mean ± SD, ^*^p≤0.05. (C) Effect of 1,25(OH)_2_D_3_ on normal oral keratinocytes. OKF6-Tert1 cells were treated with 1,25(OH)_2_D_3_ (10^−8^ M) or with 25OHD_3_ (10^−8^ M) for the indicated times. Total RNA was isolated and cyclin D1 mRNA quantified by real-time PCR. Mean ± SD, ^*^p≤0.0001.

Given the enhanced proliferation in VDR knockdown cells, we analyzed expression of cyclin D1, a key regulator of the G1-S phase of the cell cycle. Basal levels of cyclin D1 mRNA and protein were significantly higher in OKF6-VDRKO cells. Compared with control cells, VDR knockdown resulted in a 2.3-fold increase in cyclin D1 mRNA (Fig. 2A, p≤0.0001) and a 2-fold increase in the cyclin D1 protein levels (Fig. 2B, p≤0.05). Notably, these effects were observed in cells grown in the proliferative state, in the absence of 1,25(OH)_2_D_3_, suggesting that at a basal state VDR mediates cyclin D1 repression, either directly or indirectly in a ligand-independent manner. To further investigate this possibility, we examined the effect of vitamin D treatment on cyclin D1 mRNA levels. Indeed, 1,25(OH)_2_D_3_-treatment of normal OKF6-TERT1 cells decreased cyclin D1 mRNA expression approximately 1.4-fold by 6 h and 765-fold by 12 h (Fig. 2C, p≤0.05), suggesting that vitamin D-mediated signaling regulates cyclin D1 mRNA expression. Notably, treatment with 25OHD_3_ also yielded a decrease in cyclin D1 mRNA.

### VDR knockdown abrogates the response to differentiation stimuli

**Figure 3.**
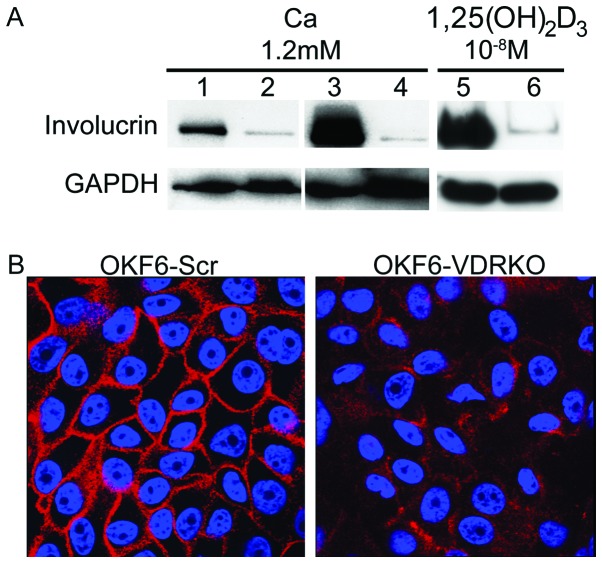
VDR silencing disrupts oral keratinocyte differentiation. (A) Expression of differentiation marker, involucrin. Cells were grown in proliferative conditions (calcium concentration 0.03 mM, lanes 1 and 2) or were stimulated to differentiate with calcium (1.2 mM for 24 h) or 1,25(OH)_2_D_3_ (10^−8^ M for 48 h). Whole cell lysates were immunoblotted to detect involucrin or GAPDH, as loading control. OKF6-Scr, lanes 1, 3 and 5; OKF6-VDRKO, lanes 2, 4 and 6. Note low involucrin levels and lack of calcium- or 1,25(OH)_2_D_3_-induced involucrin expression in VDR knockdown cells. (B) Effect of VDR silencing on calcium-induced E-cadherin translocation to the plasma membrane. Cells were grown on coverslips and induced to differentiate (calcium concentration 1.2 mM) for 24 h. E-cadherin was detected by immunofluorescence. Note decreased E-cadherin localization to intercellular junctions in OKF6-VDRKO cells.

We next examined keratinocyte differentiation in VDR knockdown cells. For these studies, cells were treated with 1,25(OH)_2_D_3_ (10^−8^ M), or with calcium (1.2 mM), a well-established regulator of keratinocyte differentiation at this concentration. To monitor differentiation, we analyzed expression of involucrin, a keratinocyte differentiation marker. In the basal, proliferative state (0.03 mM Ca), VDR knockdown markedly decreased baseline expression of involucrin, compared with control shRNA-transduced cells (Fig. 3A, lanes 1 and 2). Upon calcium- or 1,25(OH)_2_D_3_-stimulated differentiation, control cells showed a noticeable increase in the expression of involucrin, increasing almost 7.5-fold at 72 h (Fig. 3A, lanes 3 and 5). In contrast, both calcium and 1,25(OH)_2_D_3_ failed to induce involucrin in VDR knockdown cells (Fig. 3A, lanes 4 and 6).

Calcium-stimulation of keratinocytes results in formation of cell-cell contacts and the translocation of E-cadherin to these junctions (19). Thus, we examined whether VDR knockdown would alter this process. OKF6-Scr and OKF6-VDRKO cells were grown in the proliferative state (0.03 mM Ca) and then stimulated to differentiate with calcium (1.2 mM). After 24 h, E-cadherin translocation was examined by immunofluorescence. In control cells, membranous expression of E-cadherin was detected along the sites of intercellular contacts (Fig. 3B). In contrast, silencing of VDR markedly reduced E-cadherin translocation to the plasma membrane (Fig. 3B).

### Dietary vitamin D-deficiency increases oral epithelial proliferation, but does not cause epithelial hyperplasia or dysplasia

**Figure 4.**
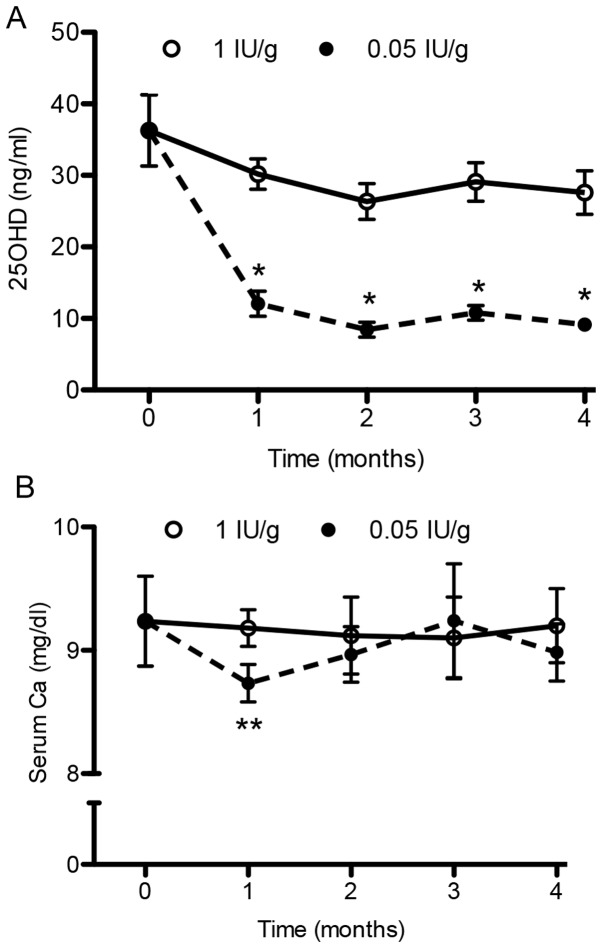
(A) Serum 25OHD_3_ concentrations, and (B) serum total calcium concentrations in mice fed with cholecalciferol replete (1 IU/g) and deficient (0.05 IU/g) diets. Mean ± SD, ^*^p≤0.0001 and ^**^p≤0.05, compared with the replete diet.

Given the effects of vitamin D signaling on oral keratinocyte proliferation *in vitro,* we examined whether modulation of dietary vitamin D altered oral keratinocyte proliferation *in vivo.* In these experiments, mice were subject to chronic dietary vitamin D deficiency. Prior to the start of dietary manipulations, the serum 25OHD concentrations ranged from 28 to 43 ng/ml (36.3±5 ng/ml, mean ± SD). One month after initiation of the experimental diets, the sedum 25OHD concentrations in mice fed with the vitamin D-deficient diet decrease significantly to 12.1±1.8 ng/ml (p<0.0001, Fig. 4A). These decreased concentrations were maintained through the remainder of the experimental period in mice fed the vitamin D-deficient diet (Fig. 4A). Through the experimental period, the serum total calcium concentrations in mice fed with the vitamin D-replete diet ranged from 8.6 to 9.5 mg/dl (9.1±0.3, mean ± SD). One month after initiation of the vitamin D-deficient diet, serum calcium concentration was slightly decreased in mice on the vitamin D-deficient diet (8.7±0.1 vs. 9.2±0.1 for the deficient and replete diets, respectively, p<0.05). However, the absolute total calcium concentrations were within the overall range of normal. Notably, through the remainder of the experimental period, there were no differences in the serum total calcium concentrations between the deficient and replete dietary groups. Overall, the biochemical changes confirmed that the vitamin D-deficient diet induced a state of chronic vitamin D deficiency.

**Figure 5.**
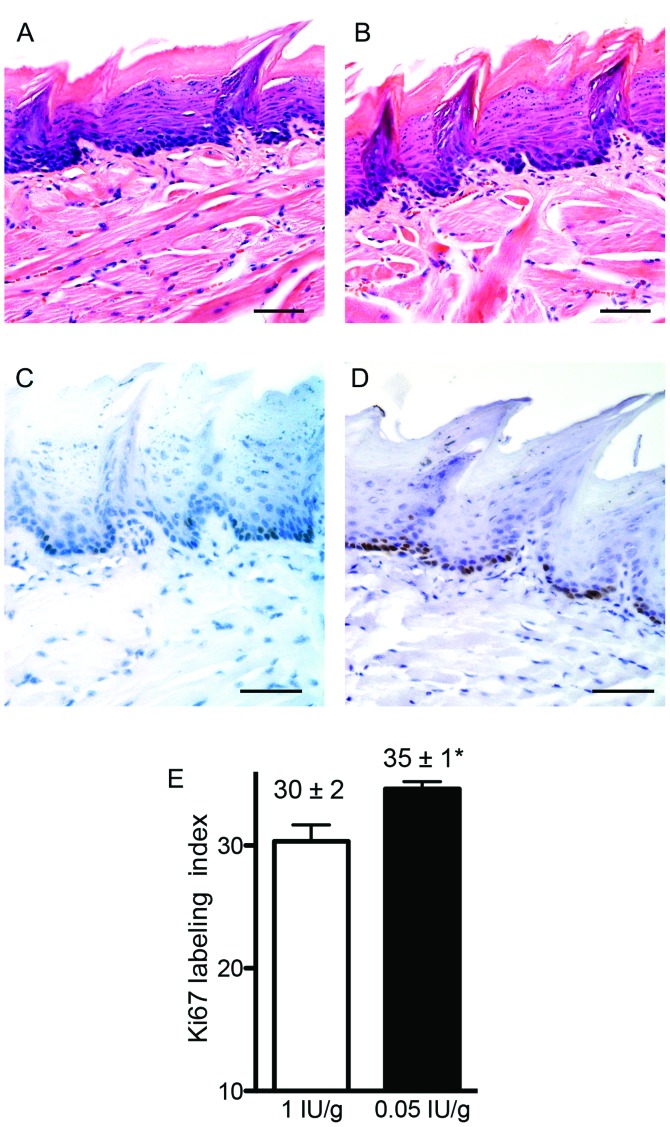
(A and B) Hematoxylin and eosin stained sections from tongue of mice fed (A) cholecalciferol replete and (B) deficient diets. Both sections demonstrate normal epithelial architecture with absence of nuclear atypia and epithelial hyperplasia dysplasia. (C–E) Ki67 immunostaining of sections from tongue of mice fed with (C) cholecalciferol replete and (D) deficient diets. (E) Quantitative Ki67 labeling index in tongue from mice fed the replete (1 IU/g) and deficient (0.05 IU/g) diets. ^*^p≤0.05. Magnification bar, 100 *μ*m.

At the end of the 4-month dietary manipulation, the oral mucosa in mice from both dietary groups was morphologically normal, and there was no evidence of any white patches or growth to suggest preneoplastic or neoplastic lesions. Histological examination of the tongues revealed normal epithelial architecture in both groups, with no evidence of nuclear atypia, epithelial dysplasia or neoplasia. Immunohistochemical detection of proliferation marker showed a similar pattern of staining in the oral epithelium from both dietary groups, with immunoreactivity being restricted predominantly to the basal cell layer (Fig. 5B). The Ki67 labeling index was increased in the epithelia of mice from the vitamin D-deficient dietary group (Fig. 5C, p<0.05). Although the magnitude of this increase was modest (approximately 16% increase), it was consistently noted in all of the vitamin D-deficient diet mice.

### Increased VDR expression in human and murine oral SCC

**Figure 6.**
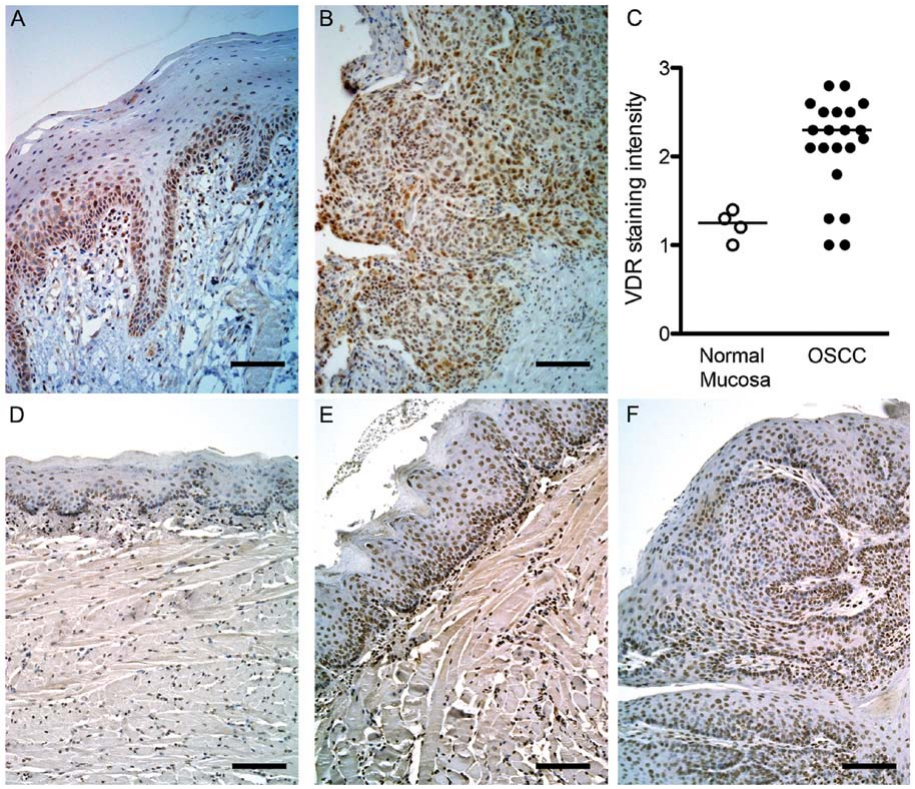
VDR immunostaining in human and murine OSCC. (A) Normal human oral mucosa showing VDR immunoreactivity predominantly in the basal layer. (B) Human OSCC showing increased VDR immunoreactivity dispersed throughout the neoplastic lesion. (C) Scatter plot showing quantitative scores of VDR immunostaining in human normal oral epithelium and human OSCC. The median score is indicated by the horizontal line. (D) Normal murine oral epithelium showing VDR immunostaining in the basal layer. Increased VDR immunoreactivity in (E) tumor-adjacent hyperplasia and (F) OSCC in mice. Magnification bars, 100 *μ*m.

Given the increased proliferation in VDR knockdown cells, as well as prior reports of deregulated epidermal homeostasis in VDR knockout mice, we examined whether VDR expression was altered in human oral SCC. In non-neoplastic oral epithelium, we detected strong nuclear VDR immunoreactivity, predominantly in the basal cell layer, with less intense and sparse expression in the suprabasal layers (Fig. 6A). In contrast, in oral SCC lesions we observed a significantly higher VDR immunoreactivity through most of the neoplastic tissue (Fig. 6B). This increased VDR staining intensity was observed in 16 of the 21 human OSCCs. The median staining intensity in OSCC was significantly higher than that in normal oral epithelium (2.3 vs. 1.3, respectively, p<0.05, Fig. 6C), suggesting deregulation of VDR expression in OSCC.

An identical pattern of altered VDR immunoreactivity was also observed in carcinogen-induced murine OSCC. Treatment with 4NQO induced severe epithelial dysplasia in all mice and caused OSCC in 3 of the 5 mice. In histologically normal oral mucosa from mice, VDR immunoreactivity was observed predominantly in the basal cell layer (Fig. 6D). Similar to human OSCC, VDR expression was increased in all murine OSCC lesions examined (Fig. 6F). Furthermore, altered VDR expression was observed in in tumor-adjacent hyperplastic/dysplastic oral epithelium, where VDR immunostaining was observed throughout the entire thickness of the epithelium (Fig. 6E).

## Discussion

The active metabolite of vitamin D, 1,25(OH)_2_D_3_, regulates proliferation and differentiation in a variety of cell types. Although these effects have been well studied in dermal keratinocytes, the role of 1,25(OH)_2_D_3_, and its mediator VDR, in regulating oral keratinocyte physiology has not been fully examined. We present data that demonstrate important roles for vitamin D signaling in oral keratinocytes, paralleling its known critical roles in dermal keratinocytes.

To investigate the role of vitamin D signaling, we generated oral keratinocytes with stable shRNA-mediated knockdown of VDR function. VDR-silencing significantly enhanced cell proliferation (Fig. 1), suggesting that VDR-mediated signaling functions to limit keratinocyte proliferation. These findings are consistent with the effects of silencing VDR in dermal keratinocytes (20) and with the 1,25(OH)_2_D_3_ effects on inhibition of cell proliferation (6). Notably in our experiments, the oral keratinocytes were grown in a serum-free medium, without 25OHD or 1,25(OH)_2_D_3_. Thus, the consequences of VDR-knockdown on cell proliferation suggest a basal, ligand-independent action of VDR to restrain cell proliferation. Nevertheless, the ability of 1,25(OH)_2_D_3_ to inhibit cell proliferation in cells with functional VDR suggests that ligand-binding potentiates the VDR basal anti-proliferative effect. VDR-knockdown increased mRNA and protein levels of cyclin D1, a key regulator of the G1-S phase of the cell cycle. Furthermore, activation of VDR signaling by 1,25(OH)2D3 decreased cyclin D1 mRNA levels (Fig. 2C). Similar findings have also been reported in dermal keratinocytes, substantiating the importance of 1,25(OH)_2_D_3_-VDR signaling in regulating cyclin D1 (5). Whether these effects reflect a direct VDR-mediated transcription control of cyclin D1 are not known. Alternatively, it is possible that VDR may regulate other signaling pathways that in turn, regulate cyclin D1 transcription. Indeed, recent evidence shows that that VDR interacts with the β-catenin pathway, which is known to activate cyclin D1 transcription (21).

VDR knockdown also decreased basal expression of the differentiation marker involucrin, and abrogated calcium- and 1,25(OH)_2_D_3_-stimulated expression of involucrin. Furthermore, in the absence of VDR, calcium stimulation failed to induce translocation of E-cadherin to the plasma membrane, paralleling similar effects in dermal keratinocytes. Both, involucrin expression and E-cadherin are important events in keratinocytes differentiation, and our data underscore the importance of VDR in regulating oral keratinocyte differentiation. Overall, our data support a critical role for vitamin D in regulating oral keratinocyte physiology, similar to its established functions in dermal keratinocytes. It would be interesting to examine the effects of VDR knockout on the oral epithelium *in vivo* and whether VDR null mice would manifest an increased susceptibility to oral carcinogenesis, similar to the increased skin tumorigenesis observed in these mice (9,10).

In this study, we examined the effects of dietary vitamin D deficiency on the oral epithelium *in vivo.* Using dietary manipulation, we were able to reliably induce a state of chronic vitamin D deficiency in mice. Notably, the decreased 25OHD concentrations paralleled the 25OHD concentrations in humans with vitamin D deficiency, underscoring the clinical relevance of our animal model. Interestingly, the dietary regime did not induce any hypocalcemia. Plausibly, the calcium content of the diet (0.5%) provides for adequate calcium absorption, despite a decreased concentration of 25OHD. Thus, potential effects of this diet can be attributed principally to decreased circulating amounts of 25OHD.

Vitamin D deficiency resulted in a modest but significant increase in oral epithelial proliferation, as determined by immunohistochemical analysis of proliferation marker Ki67 (Fig. 5C–E). However, despite the increased proliferation, these mice did not manifest any morphological or histological alterations of the oral epithelium (Fig. 5) suggesting that a decreased 25OHD status alone is not sufficient to deregulate oral epithelial homeostasis. Perhaps, the low-level increase in proliferation induced by vitamin D-deficiency may be inadequate to result in hyperplastic or dysplastic changes in the oral epithelium, at least within the 4-month duration of our study. Plausibly, prolonged states of vitamin D deficiency or more severe states of vitamin D-deficiency may deregulate proliferation sufficient to cause hyperplastic or dysplastic changes in the oral epithelium. Nevertheless, our data demonstrate for the first time that dietary vitamin D deficiency modulates proliferation of the oral epithelium *in vivo.* This finding has important implications for the pathogenesis of oral precancer and cancer. First, cross-sectional epidemiological studies have shown an association between low serum 25OHD concentration and an increased risk for OSCC development (14,15). Although such associations do not demonstrate a causal effect, our finding of vitamin D-deficiency induced increased oral epithelial proliferation raises the possibility that states of lowered 25OHD may contribute, at least in part, to the development of OSCC. We and other groups have developed animal models of oral carcinogenesis (18,22). It would be interesting to use such models to examine whether vitamin D-deficiency cooperates with chemical carcinogens or other genetic alterations to modulate OSCC risk. Second, the development of OSCC is often preceded by a clinically-detectable precancerous lesion. Such lesions manifest various signs of epithelial dysplasia. However, only a subset of such dysplastic lesions progress to OSCC, and the genetic and environmental factors that influence this transition are not well understood. To that end, evaluation of the effects of dietary vitamin D on this transition will have important implications in the management of OSCC.

We found that VDR expression was increased in human OSCC, as well as in murine chemical-carcinogen induced OSCC. Given the effects of VDR on limiting cell proliferation, as well as its putative tumor-suppressor role in the skin, as demonstrated by animal studies (9,10), this increased VDR expression in the neoplasms seems paradoxical. However, increased VDR expression has also been observed in other malignancies (23–25). VDR expression in normal oral epithelium is confined to the basal cell layer, which represents the epithelial compartment with proliferating cells. Thus, the increased VDR expression in the pre-neoplastic and neoplastic tissues may be reflective of their increased proliferative state, or perhaps a compensatory increase subsequent to the enhanced cell proliferation that drives neoplastic development. Our data do not rule out the presence of inactivating mutations in the VDR gene in these neoplasms. Although specific polymorphisms of the VDR gene have been associated with a variety of cancers (26), VDR mutations have not been reported in human neoplasms. For example, analysis of parathyroid adenomas showed no mutations in the coding regions of the VDR gene (27).

In summary, our data demonstrate a crucial role for vitamin D signaling in regulating oral keratinocyte pathophysiology. A notable and novel finding of our study is that decreased serum 25OHD concentrations enhance oral epithelial proliferation. Given the widespread vitamin D-deficiency in humans, our findings have important implications for the development and progression of human OSCC.

## Figures and Tables

**Table I.** Short-hairpin RNA clones used to silence VDR function.

TRC clone ID	Target sequence	Location on VDR transcript (GI: 340202)
TRCN0000019504	CGAAGTGTTTGGCAATGAGAT	1372–1395
TRCN0000019505	GTCATCATGTTGCGCTCCAAT	923–943
TRCN0000019506	CCTCCAGTTCGTGTGAATGAT	578–598
